# ProQ binding to small RNA RyfA promotes virulence and biofilm formation in avian pathogenic *Escherichia coli*

**DOI:** 10.1186/s13567-023-01241-2

**Published:** 2023-11-22

**Authors:** Zhongxing Wang, Rui Chen, Fufang Xia, Min Jiang, Dongyu Zhu, Yuting Zhang, Jianjun Dai, Xiangkai Zhuge

**Affiliations:** 1 Department of Nutrition and Food Hygiene, School of Public Health, Nantong University, Nantong, 226019 Jiangsu China; 2 MOE Joint International Research Laboratory of Animal Health and Food Safety, College of Veterinary Medicine, Nanjing Agricultural University, Nanjing, 210095 China

**Keywords:** APEC/ExPEC, ProQ, sRNA RyfA, virulence, biofilm formation

## Abstract

Avian pathogenic *Escherichia coli* (APEC) is a notable subpathotype of the nonhuman extraintestinal pathogenic *E. coli* (ExPEC). Recognized as an extraintestinal foodborne pathogen, the zoonotic potential of APEC/ExPEC allows for cross-host transmission via APEC-contaminated poultry meat and eggs. ProQ, an RNA binding protein, is evolutionarily conserved in *E. coli*. However, its regulatory roles in the biofilm formation and virulence of APEC/ExPEC have not been explored. In this study, *proQ* deletion in the APEC strain FY26 significantly compromised its biofilm-forming ability. Furthermore, animal tests and cellular infection experiments showed that ProQ depletion significantly attenuated APEC virulence, thereby diminishing its capacity for bloodstream infection and effective adherence to and persistence within host cells. Transcriptome analysis revealed a decrease in the transcription level of the small RNA (sRNA) RyfA in the mutant FY26Δ*proQ*, suggesting a direct interaction between the sRNA RyfA and ProQ. This interaction might indicate that sRNA RyfA is a novel ProQ-associated sRNA. Moreover, the direct binding of ProQ to the sRNA RyfA was crucial for APEC biofilm formation, pathogenicity, adhesion, and intracellular survival. In conclusion, our findings provide detailed insight into the interaction between ProQ and sRNA RyfA and deepen our understanding of the regulatory elements that dictate APEC virulence and biofilm development. Such insights are instrumental in developing strategies to counteract APEC colonization within hosts and impede APEC biofilm establishment on food surfaces.

**Supplementary Information:**

The online version contains supplementary material available at 10.1186/s13567-023-01241-2.

## Introduction

Pathogenic *Escherichia coli* are typically categorized into the following two main groups based on the clinical syndrome they cause: extraintestinal pathogenic *E. coli* (ExPEC) and intestinal pathogenic *E. coli*. ExPECs are responsible for severe extraintestinal infections in both humans and animals and can be further divided into subtypes, namely, uropathogenic *E. coli* (UPEC), neonatal meningitis *E. coli* (NMEC), avian pathogenic *E. coli* (APEC), and septicemic *E. coli* [1]. APEC strains primarily invade poultry via the respiratory tract. They bypass the immune defences of the lungs and enter the bloodstream to cause bloodstream infection (BSI) and sepsis, which can further result in multisystem infection [2–4]. Avian colibacillosis is a severe disease that significantly impacts the poultry industry, this disease manifests in chickens with symptoms such as sepsis, air sacculitis, cellulitis, salpingitis, peritonitis, encephalitis, etc. [5, 6]. In 2012, APEC was identified as a foodborne pathogen with zoonotic potential. Subsequent studies pointed to APEC as a reservoir for virulence and antibiotic resistance genes. These genes can be horizontally transferred to human ExPEC strains [7, 8].

Direct inhalation of faecal dust contaminated with APEC can trigger colibacillosis in poultry, causing severe diseases and the subsequent condemnation of carcasses [8, 9]. Notably, APECs not only persist in the intestines of healthy chickens but also might contaminate retail chickens during slaughtering from the faecal matter of infected poultry. This is probably the predominant route of APEC contamination in poultry meat products [10–12]. The manipulation or consumption of these tainted raw products can expose humans to these APEC strains [2]. Biofilms are structured communities of bacterial cells enveloped in a self-produced polymeric matrix [13, 14]. Individual cells or clusters of APEC/ExPEC can adhere to both organic and inorganic surfaces, such as waterline equipment, feeding troughs, and poultry farm grounds, and eventually form biofilms [15]. The presence of APEC/ExPEC biofilms on poultry products, food processing apparatuses, water conduits, and ventilation systems can promote bacterial transmission and spread [16]. Importantly, these biofilms safeguard APEC/ExPEC cells from antibiotic treatment. Biofilm formation significantly diminishes bacterial susceptibility to antibacterial agents, thereby leading to multi-fold increases in antibiotic resistance. This poses substantial challenges in terms of bacterial infection control [17]. To effectively eliminate these bacterial biofilms, particularly on food product surfaces, understanding the molecular mechanisms driving APEC/ExPEC biofilm formation is crucial [18–21].

APEC/ExPEC is a facultative intracellular pathogen that utilizes multiple intricate steps to establish an infection. Despite the diversity among ExPEC subtypes, these strains often share common infection strategies. Initially, fimbriae and pili facilitate *E. coli* adhesion to host cells. Following this attachment, ExPEC secretes toxins and effectors to counteract the host immune response. Subsequently, ExPEC manipulates and hijacks host cellular signalling pathways, thereby facilitating their invasion and colonization within these cells [22]. During this cascade of infection events, the expression of virulence genes in APEC/ExPEC is commonly upregulated by several DNA-binding proteins [23, 24]. Recent studies have highlighted the role of various RNA binding proteins (RBPs) in *E. coli*, such as Hfq, ProQ, and CsrA. These RBPs target mRNA transcription, modulating both their stability and translational kinetics [25–28].

*Escherichia coli* serves as a pivotal bacterial model for understanding the roles of RBPs in virulence. To date, most investigations have gravitated towards the RBP Hfq. For instance, Hfq mutant strains in *E. coli* exhibit reduced adaptability to oxidative stress and osmotic imbalance [29–31]. Hfq, a conserved RNA chaperone in *E. coli*, collaborates with sRNA to modulate various cellular activities [32]. To date, numerous Hfq-dependent sRNAs have been characterized. Recently, the functional characterization of ProQ has captured interest. ProQ is an ~ 25-kDa RNA-binding protein that interacts with RNA via its N-terminal domain (NTD) and is known to post-transcriptionally regulate bacterial virulence [33, 34]. Early research has shown the high affinity of ProQ for an RNA fragment derived from mRNA FinP [35]. ProQ has since been implicated in a myriad of cellular processes, such as bacterial virulence, proline uptake, osmotic stress response, and biofilm formation [33, 36–38]. Intriguingly, ProQ acts as an sRNA chaperone and binds to the sRNA ProP in *E. coli*. This interaction modulates the transcript levels of the sRNA ProP, which impacts the uptake of compounds such as glycine, betaine, proline, and osmoprotectants [35, 39]. In *Salmonella enterica*, the critical role of ProQ in virulence is well recognized [36]. However, the regulatory roles of ProQ in APEC/ExPEC pathogenicity remain to be elucidated.

Bacterial sRNAs serve as posttranscriptional regulators of numerous physiological processes [40]. Several sRNAs are pivotal in enabling bacterial adaptation and tolerance to microenvironmental stress, including envelope and osmotic stresses, nutrient deprivation, oxidative stress, iron deficiency, and pH fluctuations [41–43]. Specifically, the sRNA RyfA has been implicated in pathogenesis, swarming motility, and biofilm formation in *E*. *coli* [44–46]. Recently, Bessaiah et al. underscored RyfA’s significance in enhancing virulence and managing oxidative/osmotic stress responses in UPEC [47].

Our study investigates the interactions between ProQ and sRNA molecules, particularly in the context of APEC/ExPEC infection and colonization. We discerned that ProQ interacts with the sRNA RyfA, thereby bolstering virulence and biofilm formation in the APEC strain FY26. Upon ProQ deletion, both the virulence and biofilm formation abilities of the APEC strain FY26 were notably diminished. Transcriptomic analyses revealed a marked decrease in the transcription of the sRNA RyfA within the mutant FY26Δ*proQ*. Furthermore, this ProQ-RyfA interaction was vital for APEC adherence to and survival within host cells. Hence, understanding the interplay between the chaperone protein ProQ and the sRNA RyfA not only deepens our understanding of APEC/ExPEC virulence and biofilm regulation but also paves the way for devising strategies to counteract APEC colonization in vivo and prevent APEC biofilm proliferation on food product surfaces in vitro.

## Materials and methods

### Animal ethics statements

All animals utilized for this study were apparently healthy and underwent acclimatization for 7 days prior to the experiments. The animal experiments were strictly performed in accordance with the guidelines approved by the Ethical Committee of Animal Experiments of Nantong University (Permit No. S20200323-256).

### Strains, plasmids, and bacterial growth conditions

Several strains and plasmids were used in this study. Detailed information can be found in Additional file 1. The pathogenic model strain APEC FY26 (WT FY26) was utilized as the principal strain to investigate the molecular pathogenesis of APEC/ExPEC. The complete sequence of the FY26 strain (GenBank: CP101741) was reported in our prior research [48]. We generated mutant strains, termed FY26Δ*proQ* and FY26Δ*ryfA*, using the Red recombinase technique [49]. The complemented strain FY26C*proQ* was developed by introducing the pSTV28-*proQ* plasmid. Here, a DNA segment encompassing the entire *proQ* gene, along with its flanking regions (featuring the Ptac promoter and *rrnB* terminator), was fused to the medium-copy plasmid pSTV28. Similarly, the FY26C*ryfA* complemented strain was established by integrating the *ryfA* gene and its associated promoter with the pSTV28 plasmid. For ProQ expression, the *proQ* gene was tethered to the expression vector pET-28a. *E. coli* BL21 competent cells were utilized for protein expression, whereas *E. coli* DH5α cells were utilized for molecular cloning [50]. All *E. coli* strains were cultured in Luria–Bertani (LB) medium or LB agar plates at 37 °C with aeration. Selective antibiotics, namely, kanamycin (Kan; 50 µg/mL) and ampicillin (Amp; 100 µg/mL), were supplemented when necessary.

### Growth assay

A single bacterial colony was selected and inoculated into LB medium, followed by incubation at 37 °C for 8 h. After this growth period, the cells were sub-cultured into fresh LB medium until they reached the logarithmic phase (OD_600_ = 0.6). The bacterial cells were then harvested by centrifugation at 5000 × *g* for 10 min. The resultant pellet was washed three times and subsequently resuspended in phosphate-buffered saline (PBS). A 200 µL aliquot of this suspension was transferred into an Erlenmeyer flask with 20 mL of LB medium and incubated at 37 °C under constant shaking. Throughout this period, bacterial proliferation was regularly assessed by measuring the OD_600_ of the culture using a spectrophotometer. All assays were executed in triplicate for consistency.

### Biofilm formation assay

Bacterial biofilm formation and architecture were assessed using confocal laser scanning microscopy (CLSM). First, the GFP-expressing plasmid was introduced into APEC strain FY26, its mutants, and complemented strains via electroporation. These strains were then cultivated in LB medium until they reached an optical density (OD_600_) of 1.0. This culture was further diluted in fresh LB medium at a ratio of 1:1000. For biofilm development, 2.0 mL of the diluted bacterial culture was introduced into 6-well polystyrene plates, which had sterilized microscopic glass slides. Following a 24-h incubation at 37 °C, the plates underwent a thorough washing process with PBS (five times) to remove any nonadherent bacteria. The green fluorescent biofilms formed on plates were visualized by CLSM. For each slide, seven distinct regions were inspected, and three replicate slides were analysed for each strain.

Additionally, biofilm assays were performed using 96-well plates according to a previously described procedure [51]. Specifically, 200 µL of the diluted bacterial culture was seeded into each well and the samples were incubated statically at 37 °C for 24 h. After incubation, the culture medium was removed, and the wells were washed twice with PBS. The biofilms were then stained with 200 µL of crystal violet solution for 30 min. Any unbound dye was removed by washing the plates four times with PBS. Once the plates were air-dried for an hour, a solution of acetone and ethanol (in a 20:80 volume ratio) was added to solubilize the bound dye. The biofilm density was quantified by measuring the absorbance at 595 nm using an ELISA plate reader. Each bacterial strain was tested in triplicate.

### RNA isolation and quantitative real-time PCR

Total RNA was isolated from bacterial strains using a bacterial RNA kit (Omega BioTek) according to the manufacturer’s protocol. Subsequently, DNase I (Vazyme Biotech) was applied for 1 h to eliminate any genomic DNA. The transcription levels were assessed through quantitative real-time PCR (qRT-PCR) utilizing SYBR Premix Ex Taq DNA polymerase (TaKaRa) and gene-specific primers (details provided in Additional file 1). Transcriptional variances among the WT FY26, mutant, and complemented strains were computed from three independent qRT-PCR experiments. Changes in transcription were normalized to the housekeeping gene *dnaE* using the ΔΔCT method, as established in earlier studies [52, 53].

### Cell adhesion assay

To assess bacterial adhesion, a monolayer of DF-1 chicken embryo fibroblasts was infected with WT FY26, mutants, and complemented strains using a multiplicity of infection (MOI) of 100, following the protocol from a previous study [51]. After a 2-h incubation at 37 °C in a 5% CO_2_ atmosphere to facilitate bacterial adherence, the DF-1 cells were washed three times with PBS. These cells were then lysed using 1% Triton X-100. The lysates were serially diluted and plated onto LB agar plates to determine the number of adherent bacteria. The experiment was conducted in triplicate.

### Macrophage infection assays

The rates of replication of the APEC/ExPEC strains were evaluated using methods detailed in earlier studies [24, 54]. Initially, monolayer macrophages were infected with WT FY26, mutant, and complemented strains at an MOI of 10. One hour post-infection (hpi), the supernatant was discarded, and gentamicin was introduced to non-internalized bacteria. Infected cells were subsequently harvested at the following six intervals: 2, 4, 6, 8, 10, and 16 hpi. Following a PBS wash, the cells were lysed using 0.1% Triton X-100. Serial dilutions of the cell suspension were used to determine the amount of total internalized bacteria. This count was then compared with the levels from the 2 hpi time point to gauge the intracellular persistence of APEC/ExPEC. Each experiment was repeated three times, with each repetition including three biological replicates.

### Immunofluorescence imaging assay

To assess intracellular APEC abundance, immunofluorescence assays were executed based on prior methodologies [24, 54]. Monolayered HD11 cells were exposed to WT FY26, FY26Δ*proQ*, and FY26Δ*ryfA* at an MOI of 5. At 4 hpi, HD11 cells underwent washing with four times the amount of PBS, fixation in 3% paraformaldehyde for 15 min, permeabilization with 0.1% Triton X-100 and blocking with 5% BSA. Subsequently, the cells were treated with polyclonal mouse anti-APEC serum (derived from mice vaccinated with inactivated FY26 bacteria), followed by staining with FITC-conjugated goat anti-mouse IgG, phalloidin, and DAPI. Visualization and imaging were carried out using a Zeiss LSM-510 META confocal microscope. The colonization of bacterial cells was quantified in over 200 infected cells. Bacterial counts within HD11 cells were directly determined using immunofluorescence microscopy. All experiments were performed in triplicate with three biological repetitions.

### Animal experiments

Seven-day-old specific pathogen-free (SPF) chickens were utilized to elucidate the roles of ProQ and the sRNA RyfA in APEC/ExPEC virulence [55]. Briefly, chickens received an intratracheal dose of APEC strains at 5.5 × 10^5^ CFU/chicken. After infection, chicken health was assessed three times daily, and mortality was documented. By 7 days post-infection (dpi), the virulence of the bacterial strains was deduced based on observed mortality, with survival patterns analysed daily.

To delineate the roles of ProQ and RyfA in ExPEC infection in vivo, we employed a systemic infection approach using a chicken model. This allowed for the evaluation of the colonization and proliferation capacities of WT FY26, its mutant counterparts, and complemented strains within the chicken lungs, blood, liver, and spleen of infected chickens, as described previously [55]. Chickens were exposed to APEC strains at a dose of 3.0 × 10^6^ CFU/chicken. At 24 h post-infection (hpi), chickens were euthanized, and lung and blood samples were harvested. Following extraction, samples were standardized by weight, resuspended in PBS (1.0 mL/g), homogenized, serially diluted, and plated on LB agar. Colonization levels were quantified following overnight incubation.

For the murine sepsis model, 8-week-old imprinting control region (ICR) mice were subjected to intraperitoneal challenge with APEC strains at a dosage of 5.0 × 10^5^ CFU/mouse. Health monitoring continued for 7 dpi, with mortality rates signifying strain virulence. To quantify APEC lung colonization in mice, subjects received 2.0 × 10^6^ CFU/mouse of bacterial strains, and bacterial levels in mouse organs were enumerated at 24 hpi.

### Histological analyses

Organ tissues from infected chickens were promptly harvested and fixed in formalin for histological evaluation to discern the pathological alterations induced by APEC. These tissues were embedded in paraffin, sectioned (4–6 μm) using a rotary microtome, and mounted on slides. Following deparaffinization with xylol and rehydration via graded ethanol, sections were stained with haematoxylin and eosin (HE) and examined under a Nikon light microscope.

### Expression and purification of recombinant proteins

To produce the His-tag recombinant ProQ protein, recombinant plasmids were transformed into *E. coli* BL21 cells. Upon reaching an OD_600_ of 0.6 in LB medium, protein expression was initiated with 1.0 mM isopropyl-β-d-thiogalactopyranoside (IPTG) and overnight incubation at 16 °C. ProQ was subsequently isolated using Ni-chelating chromatography according to the manufacturer’s instructions. Protein concentration was quantified using the Bradford assay and a SmartSpec 3000 spectrophotometer.

### Northern blot analysis

The transcriptional levels of the sRNA RyfA in the WT FY26, the mutant, and complemented strains were assessed using northern blotting as described previously [56]. Briefly, the total bacterial RNA underwent 8% polyacrylamide urea gel electrophoresis at 300 V for 90 min. This RNA was then transferred to a Zeta-Probe GT membrane (Bio-Rad) at 20 V over 16 h, followed by UV crosslinking. The membrane was probed overnight at 45 °C with a 5′-biotin-TEG-labelled RyfA-specific probe in ULTRAhyb-Oligo Hybridization Buffer (Ambion). Bacterial 5S RNA, which was detected using a biotin-labelled probe (Additional file 1), served as an internal control. RNA bands were visualized with a GE Typhoon imaging scanner and quantified using ImageJ.

### Electrophoretic mobility shift assay (EMSA)

In vitro synthesis of sRNA RyfA was achieved using the TranscriptAid T7 High Yield Transcription Kit (Thermo Scientific) with the integral *ryfA* gene DNA template and specific primers (Additional file 1). To elucidate the binding affinity of ProQ to the sRNA RyfA, EMSA was executed using a 20 µL reaction mixture containing binding reaction buffer (100 mM NaCl, 25 nM Tris-HCl, and 1 mM EDTA; pH 7.5) [35]; the sample underwent a denaturation process at 90 °C, followed by a gradual cooling to room temperature at a rate of 2 °C/min. Post-denaturation, the mixture was incubated for 30 min at room temperature, with subsequent glycerol (20%) addition. The mixture was then resolved on a 6% (19:1) nondenaturing PAGE (25 mM Tris, 190 mM glycine, approximately pH 8.0) for approximately 2–3 h at 180 V. Northern blotting results were used to pinpoint the mobility shift of the sRNA RyfA. The nondenaturing PAGE gel contents were transferred onto a Zeta-Probe GT membrane, and sRNA RyfA detection was achieved using a 5′-biotin-TEG-labelled RyfA probe.

## Results

### Deletion of the *proQ* gene attenuated the virulence of the APEC strain FY26 in vivo

**Figure 1:**
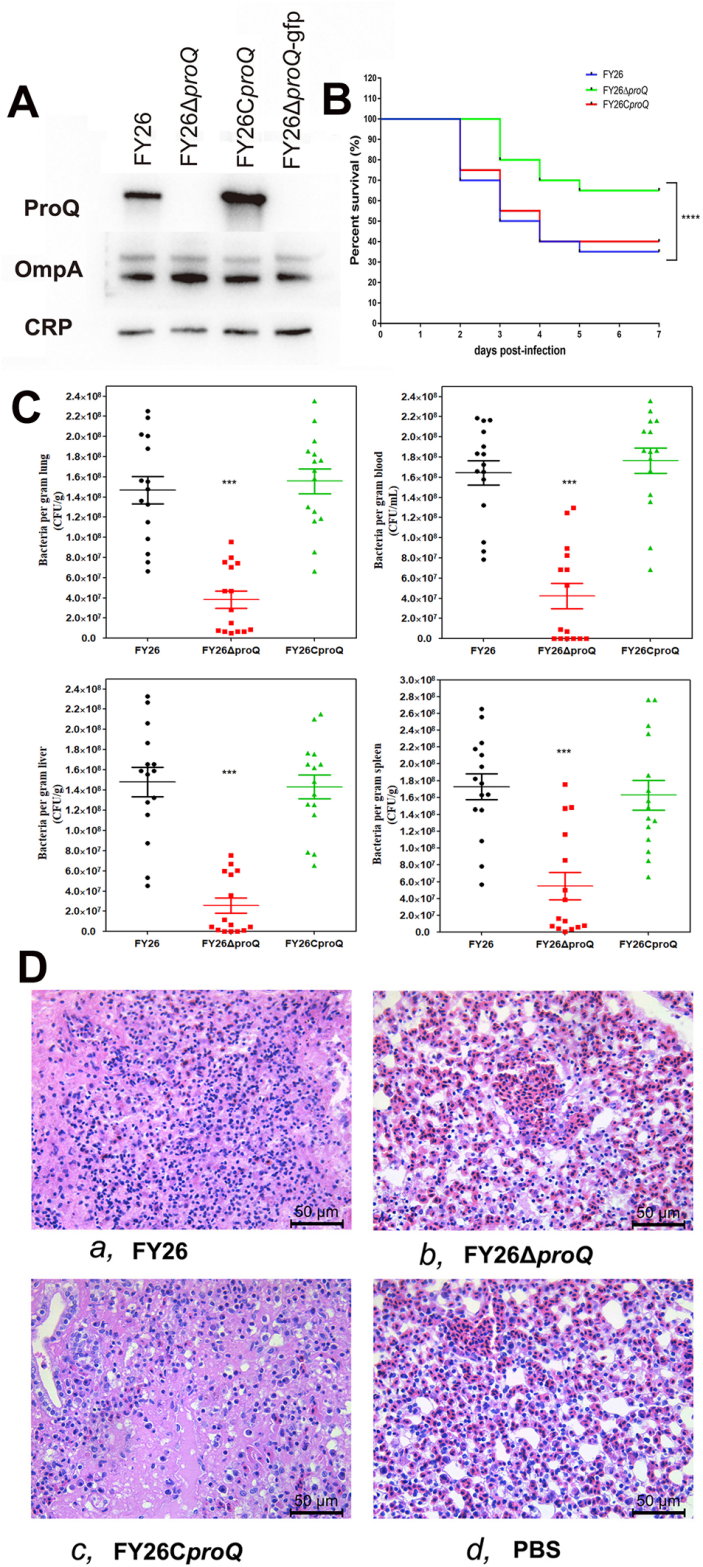
**Deletion of the *proQ *gene attenuated the virulence of the APEC strain FY26 in vivo.** **A** Western blotting analysis of ProQ expression in WT FY26, the mutant FY26Δ*proQ*, and the complemented strain FY26C*proQ*. OmpA and CRP served as reference proteins for this analysis. **B** Mortality rates of WT FY26, FY26Δ*proQ*, and FY26C*proQ* in a chicken colibacillosis model, with survival rates determined at 7 dpi. A two-way ANOVA was used to ascertain the statistical significance (*P < 0.01). **C** In vivo impact of *proQ* gene deletion on APEC colonization. A systemic infection experiment was performed in a chicken model to assess APEC proliferation in chicken lungs, liver, and spleen at 24 hpi. In addition, the proliferation of WT FY26, FY26Δ*proQ*, and FY26C*proQ* in chicken blood samples was observed. Statistical significance was determined using the nonparametric Mann‒Whitney U test. **D** Histological examination of chicken lungs following intratracheal infection with WT FY26, FY26Δ*proQ*, or FY26C*proQ* was performed. Lungs, retrieved at 24 hpi, underwent HE staining and were visualized under a light microscope to identify pathological changes. Illustrated lung lesions: **a** WT FY26, **b** FY26Δ*proQ*, and **c** FY26C*proQ*. Control: **d** PBS-inoculated chicken lung.

To elucidate ProQ’s role in APEC virulence during infection, we constructed the mutant APEC strain FY26Δ*proQ* by deleting the *proQ* gene from WT FY26. The *proQ* mutant underwent complementation by expressing *proQ+ *from the plasmid pSTV28-*proQ*. This *proQ* gene was placed under the transcriptional control of the *Ptac* promoter and the *rrnB* terminator. For the construction of the ProQ expression plasmid, the DNA fragment, which incorporated the complete *proQ* gene alongside upstream/downstream DNA (with the artificially added Ptac promoter and *rrnB* terminator sequence), was inserted into the complemented plasmid pSTV28. Figure 1A demonstrates that ProQ expression was observed in WT FY26 and the complemented strain FY26C*proQ* but was absent in the mutant strain. The effects of ProQ on APEC growth were also examined in vitro. There was no discernible impact of *proQ* deletion on the growth of APEC strain FY26 in LB medium under regular culture conditions (data not shown).

To determine the impact of ProQ on APEC virulence, we assessed the lethality of WT FY26, the mutant FY26Δ*proQ*, and complemented FY26C*proQ* strains using a chicken colibacillosis model. Chickens were intratracheally infected with the corresponding bacterial strains at a concentration of 5.5 × 10^5^ CFU/chicken, an infectious dose mirroring the LD_50_ of WT FY26. Survival curves highlighted a notable reduction in mortality among chickens infected with FY26Δ*proQ*. The time of peak mortality in the FY26Δ*proQ*-infected group was distinctly postponed compared to that in the WT FY26-infected group (Figure 1B). The complemented strain FY26C*proQ* displayed a resurgence in virulence that aligned with that of WT FY26. These findings suggest that ProQ absence diminishes APEC/ExPEC virulence in vivo.

We also investigated the contribution of ProQ to APEC colonization in vivo by subjecting chickens to systemic infection through intratracheal inoculation with APEC strains. The set infectious dose was 3.0 × 10^6^ CFU/chicken, mirroring the LD_90_ of WT FY26. Bacterial colonization levels within chicken lungs, liver, and spleen were assessed at 24 hpi. The mutant strain FY26Δ*proQ* showed significantly compromised colonization in the lungs, liver, and spleen (Figure 1C). Relative to WT FY26-infected chickens, only approximately 60% of those infected with FY26Δ*proQ* displayed bacteraemia symptoms. Furthermore, the replication proficiency of the mutant strain FY26Δ*proQ* was decreased in chicken blood.

Concurrently, we established an infection cohort specifically for pathological assessments. Post-infection with an LD_90_ dose, chickens from each cohort were necropsied at both 24 and 48 hpi to inspect clinical presentation and organ-specific lesions, particularly focusing on air sacs, pericarditis, and perihepatitis. As delineated in Additional files 2 and 3, no observable lesions related to air sacs, pericarditis, or perihepatitis were identified at either the 24 hpi or 48 hpi time points. We postulate that the virulence of APEC strains, specifically WT FY26 and the complemented strain FY26C*proQ*, might induce a septicaemic response in chicks, resulting in mortality. Furthermore, the absence of air sac, pericarditis, and perihepatitis lesions in chickens infected with the mutant strain suggests a diminished pathogenic potential. Furthermore, manifestations typical of colibacillosis-linked lesions in air sacs, pericarditis, and perihepatitis might not be discernible during such early post-infection intervals. Histopathological evaluations at 24 hpi (Figure 1D) revealed evident lesions in chickens infected with WT FY26 and FY26C*proQ*, and the phenotypes included widened alveolar septa, congestion, and notable inflammatory cell infiltration. In contrast, FY26Δ*proQ*-infected chicken lungs showed minimal cell wall thickening and reduced inflammatory exudation. These findings highlight the essential role of ProQ in APEC/ExPEC colonization in vivo, hinting that deletion of the *proQ* gene might undermine the ability of APEC/ExPEC to overcome the immune defences in chicken lung tissue and subsequently infiltrate the bloodstream.

### ProQ played an essential role in APEC/ExPEC bloodstream infection

**Figure 2:**
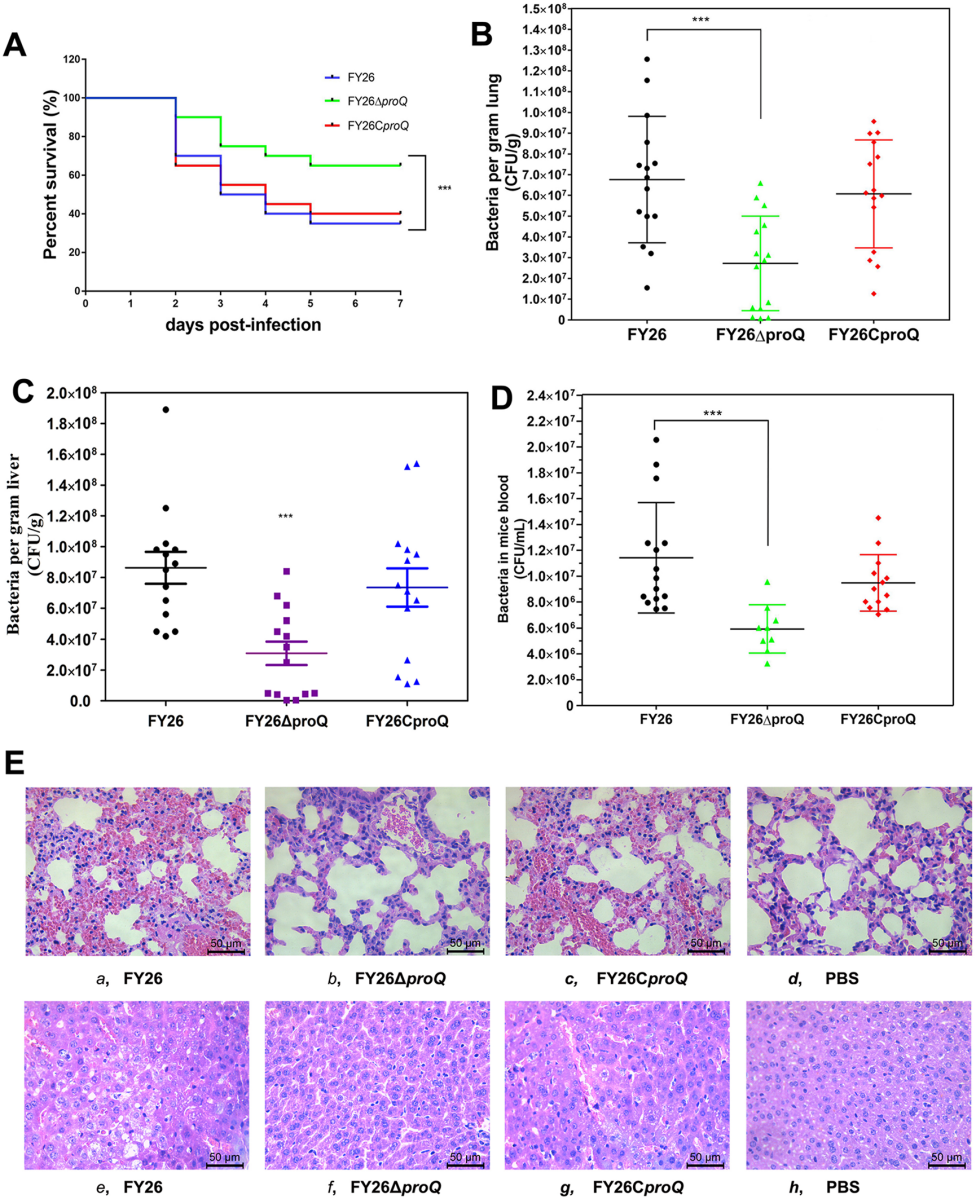
**Contribution of ProQ to APEC/ExPEC bloodstream infection.** **A** Impact of *proQ* deletion on APEC/ExPEC bloodstream infection in a murine sepsis model. Mouse survival rates were assessed at 7 dpi following infection with WT FY26, mutant FY26Δ*proQ*, or FY26C*proQ*. A two-way ANOVA was used to evaluate the significant differences. **B** Influence of *proQ* deletion on in vivo APEC/ExPEC colonization. A systemic infection study was utilized measured APEC/ExPEC proliferation in mouse lungs at 24 hpi. Statistical significance was determined using the nonparametric Mann‒Whitney U test. **C** APEC/ExPEC growth in the mouse liver observed at 24 hpi. **D** The proliferation of WT FY26, FY26Δ*proQ*, and FY26C*proQ* in murine blood samples was compared. **E** Histopathological examination of the lungs and liver of mice intraperitoneally infected with WT FY26, FY26Δ*proQ*, or FY26C*proQ*. Tissues retrieved at 24 hpi were subjected to HE staining. Illustrated lung lesions from mice are **a** WT FY26, **b** FY26Δ*proQ*, and **c** FY26C*proQ*. Control: **d** PBS-inoculated mouse lung. Depicted liver lesions from mice are **e** WT FY26, **f** FY26Δ*proQ*, and **g** FY26C*proQ*. Control: **h** PBS-inoculated mouse liver.

A previous study revealed that the APEC strain FY26 could cause sepsis and demonstrated its zoonotic potential. Notably, FY26 often serves as a positive control in discerning the zoonotic potential of isolates [57]. In this study, we sought to understand the impact of *proQ* deletion on APEC/ExPEC bloodstream infections (BSI). Using a murine sepsis model, we analysed the survival rates of mice post-infection with WT FY26, FY26Δ*proQ*, and FY26C*proQ*. Interestingly, the survival curves indicated a pronounced delay in the timing of peak mortality for mice infected with FY26Δ*proQ* when compared with their WT FY26-infected counterparts (Figure 2A). Additionally, the mutant FY26Δ*proQ* had reduced colonization efficiency in both the mouse lungs and liver, especially when compared with that of WT FY26 and the complemented strain (*P* < 0.01) (Figures 2B and C). Remarkably, only 60% of the mice infected with FY26Δ*proQ* exhibited signs of bacterial proliferation in their bloodstream (*P* < 0.01) (Figure 2D). This evidence suggests that ProQ’s absence hampers the ability of APEC/ExPEC to trigger BSI.

Pathological assessments of lung and liver tissues from APEC/ExPEC-infected mice underscored the symptoms that of characteristic of bacteraemia and sepsis. In mice infected with WT FY26, hepatocytes showed significant nuclear degradation, characterized by nuclear shrinkage, fragmentation, and disappearance. Moreover, severe pulmonary lesions were discernible in WT FY26-infected mice at 24 hpi (Figure 2E), with conspicuous widening of intervals between pulmonary alveolus in the lung lumen. In comparison, FY26Δ*proQ*-infected mice presented with localized congestion and relatively modest changes in lung pathology (Figure 2E). However, the lungs of PBS-inoculated mice remained largely unscathed and devoid of inflammatory cell infiltration. These observations confirmed that *proQ* deletion mitigated lesion progression in infected mouse lungs at 24 hpi, especially when benchmarked against the changes seen in WT FY26-infected mice (Figure 2E).

### ProQ promoted APEC/ExPEC biofilm formation

**Figure 3:**
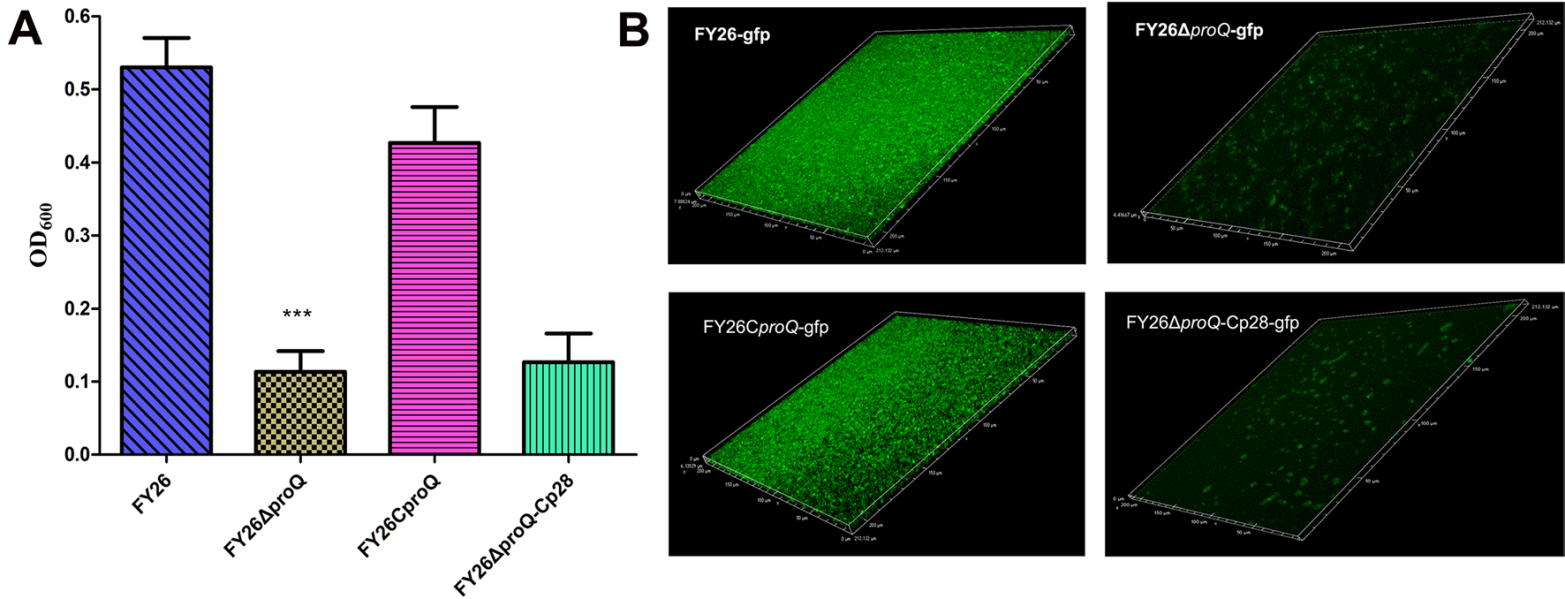
**Essential role of ProQ in APEC/ExPEC biofilm formation.** **A** Assessment of biofilm formation using the crystal violet assay for WT FY26, the mutant FY26Δ*proQ*, the complemented strain FY26C*proQ*, and the control FY26Δ*proQ-*Cp28. Strains were propagated in LB medium supplemented with 5 g/L glucose, and biofilm formation was quantified using polystyrene microtiter plates. **B** Visualization of biofilms formed by WT FY26, FY26Δ*proQ*, and FY26C*proQ* using confocal laser scanning microscopy (CLSM). Cells are represented by green fluorescence.

The biofilm formation ability of WT FY26, FY26Δ*proQ*, and FY26C*proQ* was quantitatively evaluated using a crystal violet assay. WT FY26 displayed a significantly increased biofilm density in comparison to that of the negative control strain MG1655 (*P* < 0.01), as illustrated in Figure 3A. A significant reduction in biofilm formation capacity was observed in the FY26∆*proQ* mutant when compared with WT FY26 (*P* < 0.01). Conversely, the complemented strain FY26C*proQ* exhibited a rejuvenation of biofilm formation capacity upon in-trans expression of the *proQ* gene (Figure 3A). Further insights gleaned from the CLSM analysis revealed a more diffuse biofilm structure in FY26Δ*proQ* (Figure 3B), whereas FY26C*proQ* showed a similar recovery in biofilm density relative to that of WT FY26. Collectively, these findings highlight the pivotal role of ProQ in regulating biofilm formation in APEC/ExPEC strains. However, the exact molecular mechanism by which ProQ influences biofilm formation remains to be elucidated.

### ProQ contributed to APEC/ExPEC replication within macrophages

**Figure 4:**
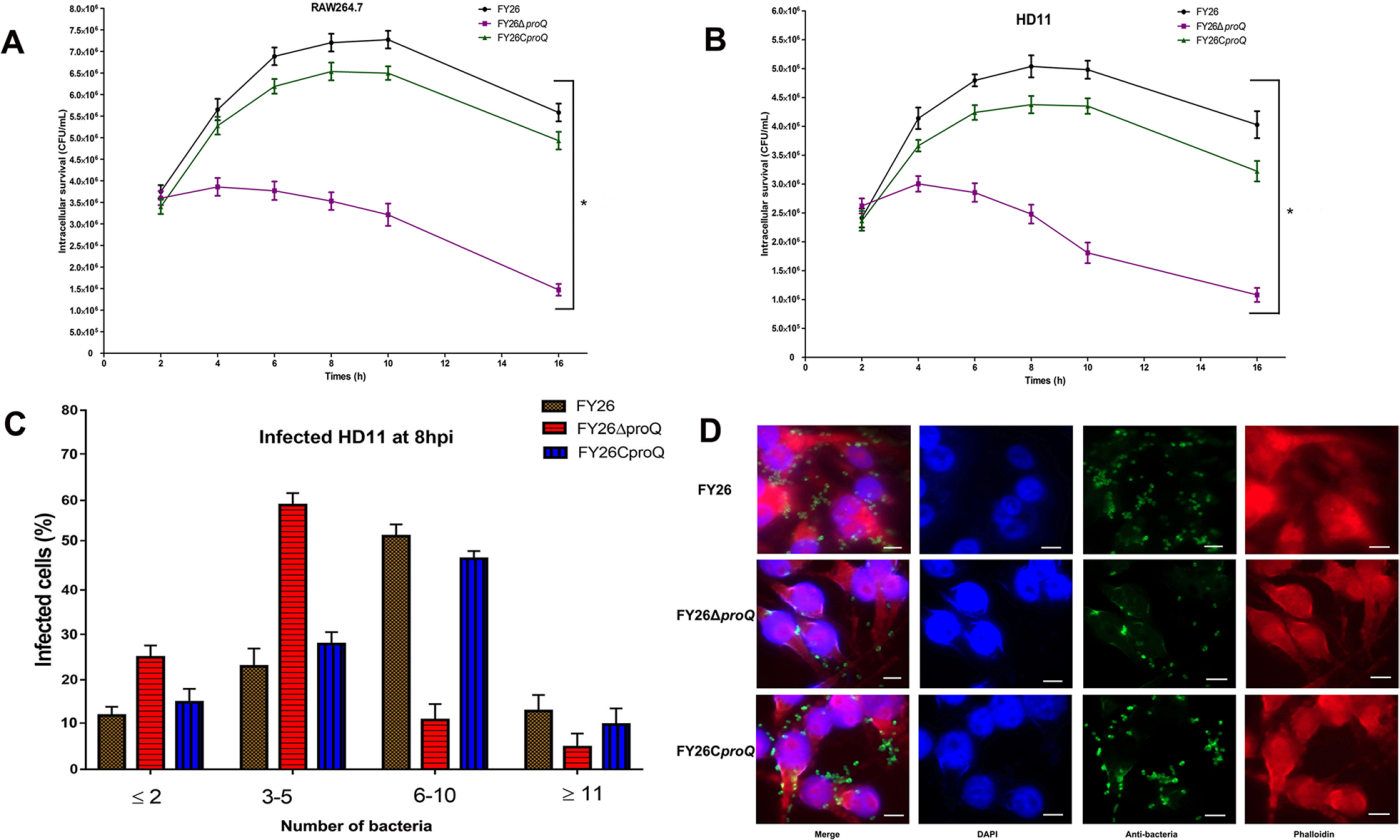
**Role of ProQ in APEC/ExPEC replication within macrophages.** **A** The intracellular replication and survival of WT FY26 cells in RAW264.7 macrophages. The data represent the cumulative results from at least four individual experiments. Significant differences were analysed using two-way ANOVA (*P < 0.01). **B** Intracellular replication and survival of mutant FY26Δ*proQ* within HD11 macrophages. **C** Evaluation of the impact of *proQ* deletion on APEC intracellular proliferation in HD11 cells was determined using an immunofluorescence assay. Quantitative data for bacterial proliferation were derived from approximately 200 infected cells across at least three independent experiments. **D** Comparative intracellular proliferation of WT FY26, FY26Δ*proQ*, and FY26C*proQ* strains in HD11 macrophages assessed at 8 hpi. Infected HD11 cells were fixed with DAPI and labelled with phalloidin. Bacteria within the cells were targeted with anti-FY26 antibody followed by labelling with FITC-conjugated secondary antibody. Scale bar represents 10 μm.

Previous studies have shown that APEC/ExPEC can proliferate and persist inside macrophages, which is a critical phase in the systemic dissemination of APEC/ExPEC [58, 59]. Figure 4A illustrates the markedly diminished replication capability of FY26Δ*proQ* in RAW264.7 macrophages. Notably, the percent survival of FY26Δ*proQ* dropped to 68.7% at 4 hpi and further to 48.7% at 8 hpi; there was a significant contrast between the survival rates seen with this strain and with the WT FY26 strain (*P* < 0.01). Similarly, the percent survival of FY26Δ*proQ* within HD11 macrophages was prominently compromised (Figure 4B). The rate of FY26ΔproQ survival dropped to 71.0% at 4 hpi and 48.6% at 8 hpi, which was a marked difference in comparison to that of WT FY26 (*P* < 0.01). Meanwhile, in the complemented strain FY26CproQ, the replication and survival capability was restored.

Subsequently, an immunofluorescence imaging assay was employed to determine the role of ProQ in APEC proliferation in HD11 macrophages. At the outset (2 hpi), over 98% of the infected HD11 macrophages presented with the WT FY26, mutant, and complemented strains. Based on the bacterial count in HD11 cells at various intervals, we created the following four categories: ≤ 2, 3–5, 6–10, and ≥ 11. The bacterial counts for WT FY26, FY26Δ*proQ*, and FY26C*proQ* in 200 infected HD11 cells at 2 and 4 hpi revealed no distinguishable variations (all ≤ 5). However, by 8 hpi, approximately 65% of WT FY26-infected HD11 macrophages contained bacterial counts of ≥ 6, with approximately 52% falling in the 6–10 bracket (Figure 4C). In stark contrast, nearly 84% of the FY26Δ*proQ*-infected HD11 macrophages harboured counts of ≤ 6, and approximately 59% ranged between 3 and 5 at 8 hpi. When compared to WT FY26-infected HD11 macrophages, a significant reduction to approximately 49% was observed in FY26Δ*proQ*-infected HD11 macrophages with a bacterial number ≥ 6 at 8 hpi. In contrast, the replication and survival of FY26C*proQ* in macrophages were largely akin to those of WT FY26. Figure 4D depicts the replication patterns of various APEC strains within HD11 macrophages at 8 hpi. Taken together, these findings underscore the critical role of ProQ in promoting APEC/ExPEC replication and persistence in phagocytic cells.

### ProQ promoted the adhesion of APEC/ExPEC to nonphagocytic cells

**Figure 5:**
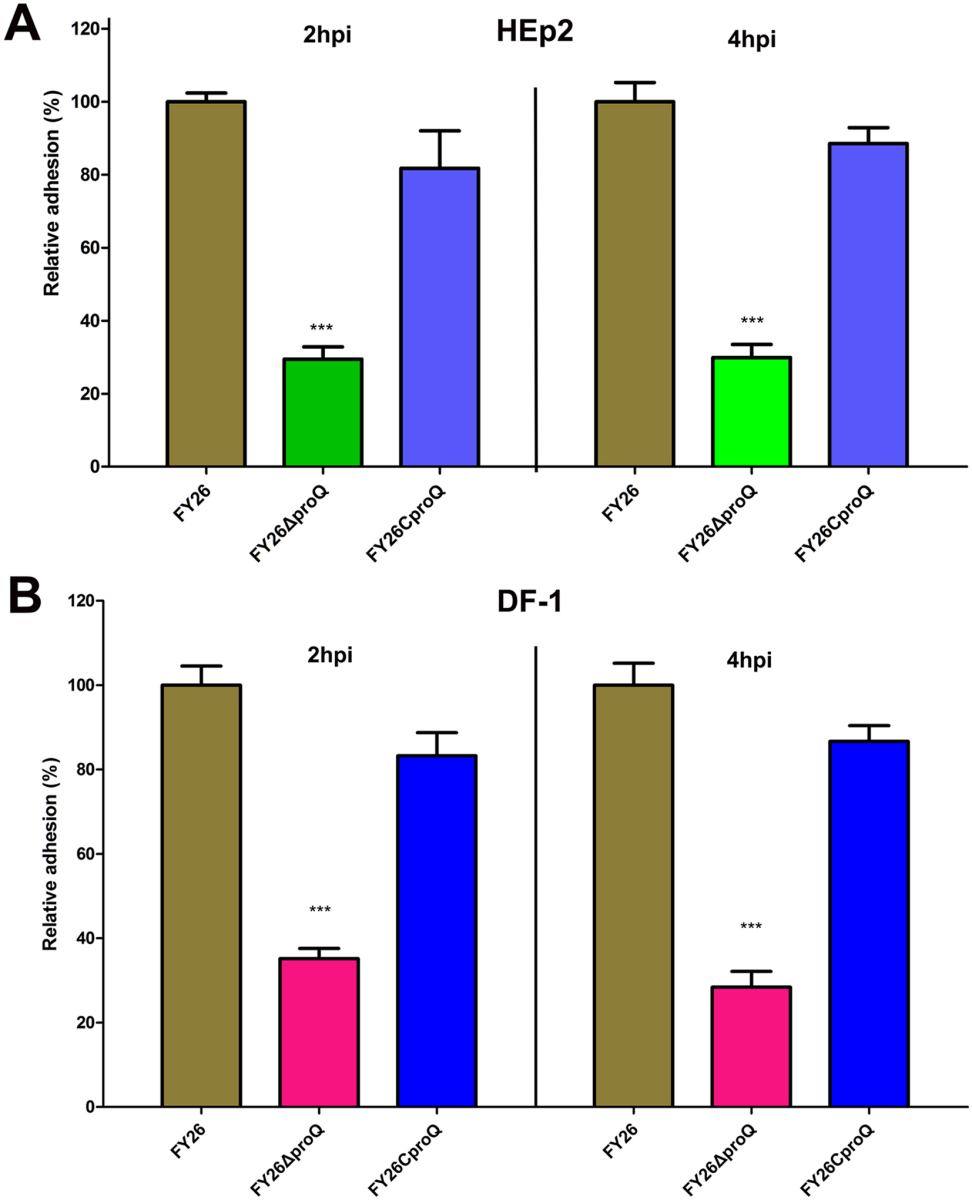
**ProQ promoted APEC/ExPEC adhesion to nonphagocytic cells.** **A** The adhesion profiles of WT FY26, the mutant FY26Δ*proQ*, and the complemented strain FY26C*proQ* to HEp2 cells, assessed at 2 and 4 hpi. The presented data are the mean values derived from three independent experiments. A significant difference was observed between WT FY26 and FY26Δ*proQ*, with *P* < 0.01. **B** The adhesion of WT FY26, the FY26Δ*proQ* mutant, and the FY26C*proQ* complemented strain to DF-1 cells was compared.

To elucidate the influence of ProQ on APEC/ExPEC adhesion to nonphagocytic cells, we conducted an in vitro adhesion assay utilizing both HEp2 cells and the chicken fibroblast line DF-1. As shown in Figure 5A, compared to WT FY26, a conspicuous reduction of approximately 70% was observed in the adherence of FY26Δ*proQ* to HEp2 cells at 2 and 4 hpi (*P* < 0.01). Parallel observations were discerned when evaluating FY26ΔproQ adherence to DF-1 cells (Figure 5B). Here, the adhesion rate of the mutant strain diminished to approximately 36.1% at 2 hpi and further decreased to approximately 31.3% at 4 hpi when compared to WT FY26 (*P* < 0.01). Notably, the complemented strain FY26C*proQ* demonstrated an adhesive capacity mirroring that of WT FY26. The pronounced attenuation in the adhesion of FY26∆*proQ* to nonphagocytic cells (Hep2 and DF-1) accentuates ProQ’s essential role in facilitating APEC/ExPEC attachment to host cells.

### ProQ affected the expression of genes involved in membrane remodelling, metabolism, and adaptation to the environment

**Figure 6:**
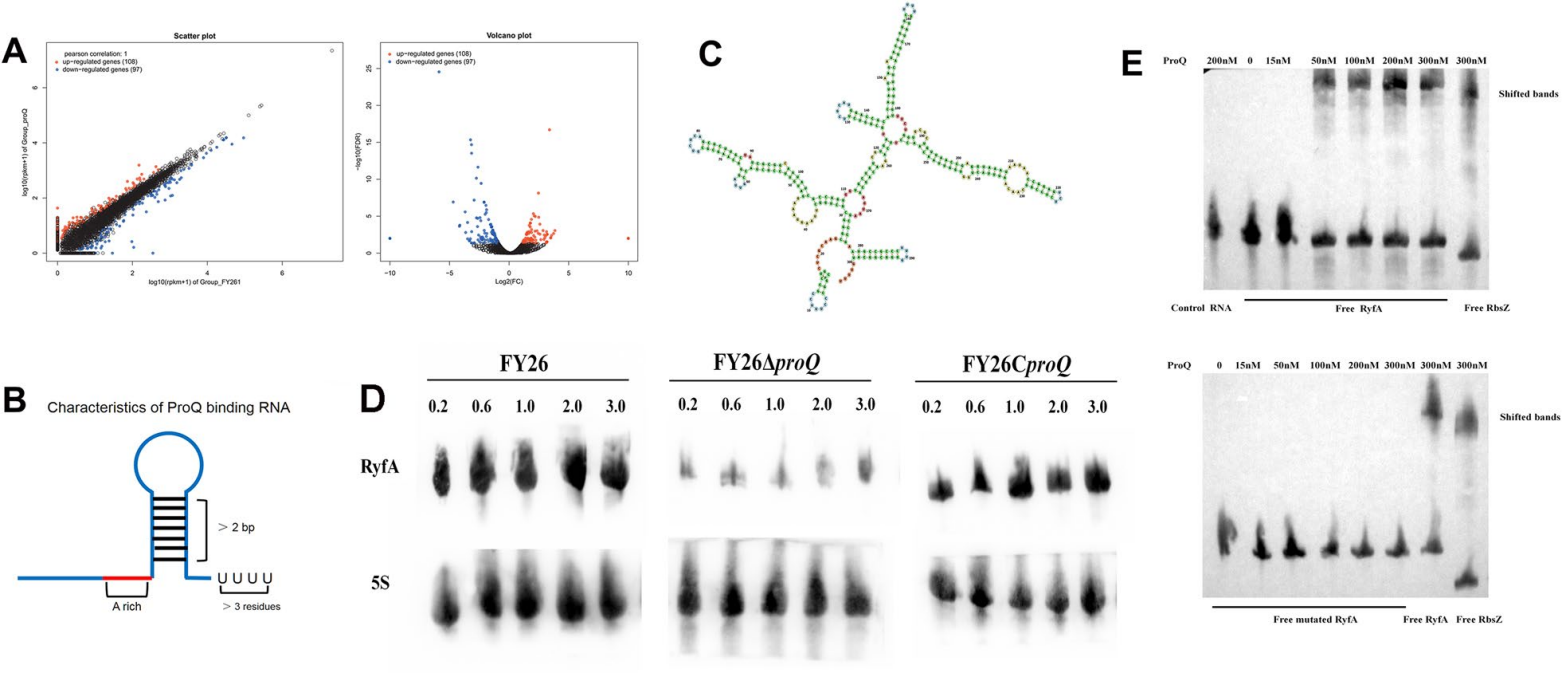
**Direct interaction of ProQ with the sRNA RyfA.** **A** The transcription profiles of WT FY26 and the mutant FY26Δ*proQ* were compared. **B** Canonical RNA structure associated with ProQ binding at intrinsic terminators, as detailed in [33]. **C** Predicted secondary structure of RyfA sRNA. **D** Temporal Northern blotting assessment of RyfA transcription across various growth stages in LB medium at 37 °C of WT FY26, FY26Δ*ryfA*, and FY26C*ryfA*. **E** Validation of the interaction between ProQ and the sRNA RyfA through EMSA. Notably, no interaction was observed between ProQ and the RyfA variant lacking a poly(U) tail, even as the ProQ concentration increased. The binding of sRNA RbsZ to ProQ served as a positive control.

To elucidate the impact of ProQ on the global transcription of APEC/ExPEC, we performed an RNA-Seq comparison of the WT FY26 and FY26Δ*proQ* strains (BioProject Accession No. SUB10505774). We extracted total RNA from bacterial cells during mid-logarithmic growth and subsequently constructed cDNA libraries. Using HiSeq4000 for sequencing, single-end reads were mapped to the APEC complete genome with the TopHat program. The overarching expression profile of the APEC genome was quantified via edgeR software. Genes with differential expression (DEGs) were pinpointed using a threshold criterion of log_2_FC ≥ 1 (*P* < 0.05). The constructed volcano plot unveiled 205 significantly differentially expressed genes (with a ≥ twofold change) between WT FY26 and FY26Δ*proQ* (refer to Figure 6A; exhaustive details about these 205 genes can be found in Additional file 4). Notably, 108 genes exhibited elevated expression in FY26Δ*proQ*. This set included genes such as *yeeE* and *ppnP*, which encode bacterial membrane proteins. Furthermore, a cluster of genes associated with sulfate transporter subunits, namely, *cysA*, *cysW*, *cysT*, *cysP*, *cysN*, and *cysD*—had over threefold increases in expression. Conversely, the expression of 97 genes, including several membrane-associated genes, such as *ompC*, *mltD*, *yagU*, *ompW*, and *ompX*, was downregulated. Notably, the *ompC*, *ompW*, and *ompX* genes encode outer membrane proteins. The list of DEGs also included *cspE* and *cspD*, which are responsible for encoding cold-shock proteins, and *hspQ*, which encodes a heat shock protein. The *glcE* and *glcD* genes, which are affiliated with FAD-binding proteins, had a more than fourfold decrease. Strikingly, the level of the sRNA RyfA decreased more than 8-fold in the mutant FY26Δ*proQ*. This prompted a thorough investigation into the relationship between the sRNA RyfA and ProQ.

### roQ directly interacted with the sRNA RyfA

Previous studies have indicated that ProQ possesses specificity in bacterial RNA binding [33]. Figure 6B demonstrates that the majority of the ProQ binding sites, as identified via in vivo crosslinking, were predominantly situated at the 3′-end of RNA and consistently overlapped with the internal terminator. Additionally, this 3′-end consistently featured a stable stem‒loop structure, appended with a single-stranded poly(U) tail with a minimum length of four nucleotides [33]. Given its secondary structure, sRNA RyfA meets the prerequisites for ProQ’s direct binding, implying a direct interaction between ProQ and sRNA RyfA (Figure 6C). To corroborate the differentially expressed genes identified in the RNA-seq analysis, RT-PCR was conducted for select genes in the WT FY26 and the mutant FY26Δ*proQ.* The qRT-PCR data mirrored the shifts in RyfA transcription between WT FY26 and FY26*ΔproQ*, as was initially shown by the RNA-seq results (data not shown). Northern blotting investigations further illuminated a marked reduction in RyfA transcriptional levels in FY26Δ*proQ* cells in various growth phases when compared with that of WT FY26 cells. In stark contrast, transcription was undetectable in the RyfA deletion mutant FY26Δ*ryfA* (Figure 6D). This set of data emphasizes the role of ProQ in enhancing the transcription of sRNA RyfA within the APEC strain FY26; deletion of the proQ gene leads to a pronounced decrease in RyfA transcription. EMSAs showcasing the binding of ProQ to the sRNA RyfA displayed more intense shifted band, concomitant with a reduction in the intensity of the lower band as the concentration of ProQ increased (Figure 6E). These observations point to an increasing proportion of RyfA associating with ProQ, thereby leading to a larger ribonucleoprotein complex with diminished electromobility. Predictably, the mutated RyfA, devoid of a poly(U) tail, displayed no migration (Figure 6F). In conclusion, ProQ seems to directly interact with the sRNA RyfA, thereby enhancing its transcriptional dynamics.

### ProQ promoted APEC/ExPEC biofilm formation by promoting RyfA transcription

**Figure 7:**
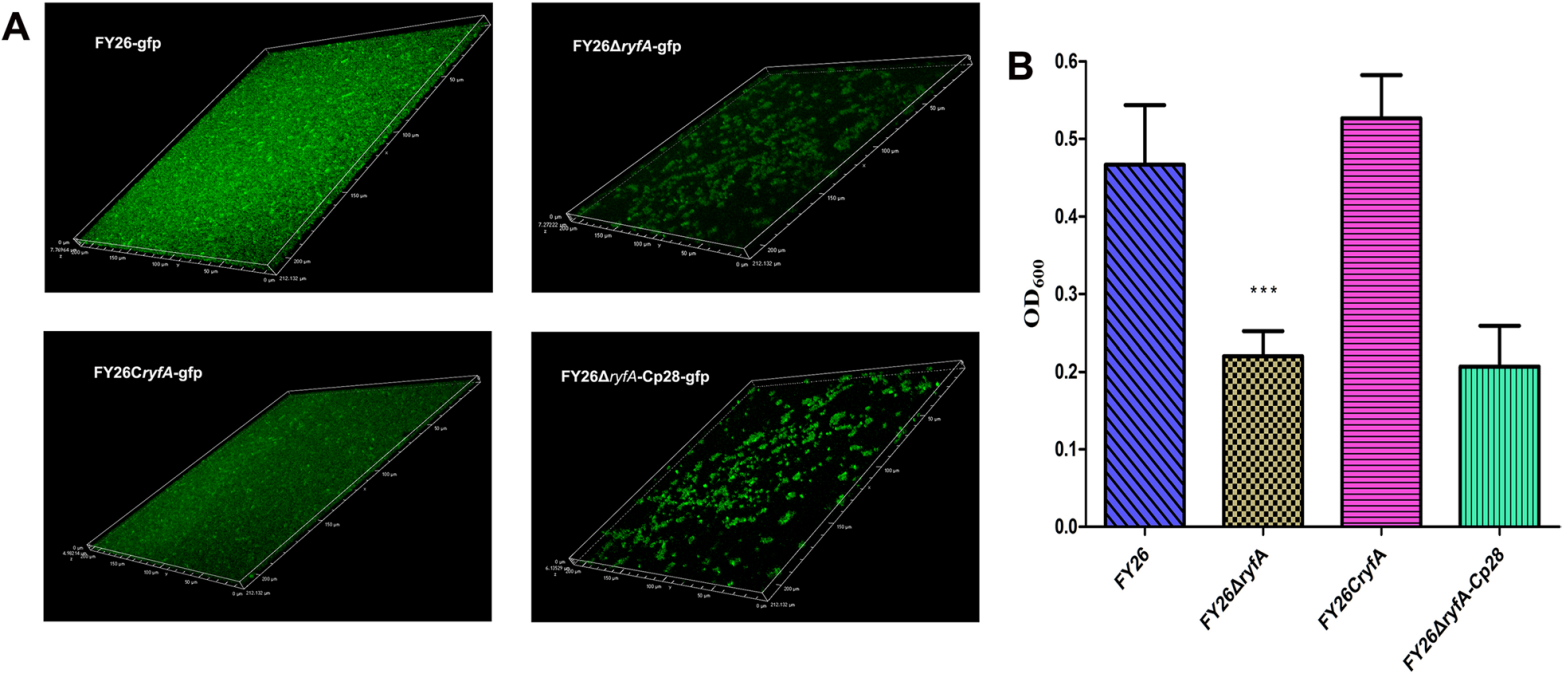
**RyfA promoted APEC/ExPEC biofilm formation.** **A** CLSM visualization of biofilms formed by WT FY26, FY26Δ*ryfA*, and FY26C*ryfA*, which all exhibited green fluorescence. **B** Assessment of the biofilm formation capabilities of WT FY26, FY26Δ*ryfA*, and FY26C*ryfA* and the negative control strain MG1655 using a crystal violet assay. APEC strains were grown in LB medium supplemented with 5 g/L glucose.

Given the role of ProQ in promoting the transcriptional stability of RyfA, we investigated the impact of *ryfA* deletion on APEC biofilm formation. CLSM analysis revealed notably stronger GFP fluorescence intensity in the WT FY26 biofilm than in the mutant FY26Δ*ryfA* biofilm (Figure 7A). The impaired biofilm formation was partially restored in the complemented strain FY26C*ryfA*. However, the FY26Δ*ryfA* mutant biofilm had a considerably weaker and more disintegrated fluorescence signal than the FY26Δ*proQ* mutant biofilm (Figure 7A). Using crystal violet staining to quantify the biofilm formation of the WT FY26, FY26Δ*ryfA*, and FY26C*ryfA* strains, the outcomes aligned with the observations from CLSM analysis (Figure 7B). Collectively, these results suggest that RyfA plays a pivotal role in facilitating APEC biofilm formation. Moreover, the interaction between ProQ and the sRNA RyfA is a vital determinant of the ability of APEC/ExPEC to form biofilms.

### The sRNA RyfA impacted APEC/ExPEC virulence

Given the established connection between ProQ and the sRNA RyfA, we sought to determine the significance of RyfA in APEC/ExPEC virulence. The virulence attributes of WT FY26, mutant FY26Δ*ryfA*, and the complemented strain FY26C*ryfA*, including adhesion and intracellular survival, were scrutinized in both chicken colibacillosis and mouse sepsis models. In chicken models, infection with the mutant FY26Δ*ryfA* led a notably diminished mortality rate when compared with that of WT FY26 infection (Additional file 5A). Similarly, chickens infected with this mutant had a delayed mortality peak. The colonization capacity of the mutant in chicken lungs, liver, and spleen was markedly diminished in comparison to that of WT FY26 (Additional file 5B). Correspondingly, the pathological lung alterations in chickens instigated by FY26Δ*ryfA* were less severe than those instigated by WT FY26 (Additional file 5C). These findings underscore the indispensability of the sRNA RyfA for APEC/ExPEC colonization of chicken lungs. Mice inoculated with FY26Δ*ryfA* displayed a delayed mortality peak when compared to that of mice infected with WT FY26 (Additional file 5D). The colonization and pathogenic potential of the mutant strain within the lungs and liver of mice were markedly reduced (Additional file 5E, F) (*P* < 0.01), suggesting that the sRNA RyfA contributes to APEC/ExPEC bloodstream infections.

Furthermore, in vitro assays revealed that FY26Δ*ryfA* had compromised adhesion to nonphagocytic HEp2 and DF-1 cells. Relative to WT FY26, the mutant’s adherence to HEp2 cells was reduced to 48.1% and 51.6% at 2 and 4 hpi, respectively (*P* < 0.01) (Additional file 6A). Comparable outcomes were observed in terms of adhesion to DF-1 cells (Additional file 6B). The adherence capacity of the complemented strain, FY26C*ryfA*, paralleled that of WT FY26.

Moreover, FY26Δ*ryfA* exhibited diminished replication in RAW264.7 macrophages. The survival rate of FY26Δ*ryfA* at 4 and 8 hpi were merely 73.8% and 49.1% that of the WT FY26, respectively (*P* < 0.01) (Additional file 6C). Likewise, the survival rate of FY26Δ*ryfA* in HD11 macrophages was reduced to approximately 78.9% and 56% of that of WT FY26 at 4 and 8 hpi, respectively (*P* < 0.01) (Additional file 6D). Immunofluorescence data indicated that only approximately 31.7% of FY26Δ*ryfA*-infected macrophages contained bacteria counts ≥ 6 at 8 hpi (Additional file 6D), and the survival of all strains in HD11 macrophages at 8 hpi is detailed in Additional file 6E. In summary, these results signify that the sRNA RyfA is pivotal for APEC/ExPEC replication and survival in macrophages. Furthermore, ProQ’s interaction with and upregulation of sRNA RyfA transcription underpin successful APEC/ExPEC infection in macrophages.

## Discussion

In recent years, sepsis caused by ExPEC BSIs has been on the rise and poses a significant threat to public health [60]. APEC/ExPEC, recognized as an extraintestinal foodborne pathogen, has close ties with ExPEC isolates that are responsible for human BSI [61–63]. Complicating matters further is the emergence of multidrug-resistant APEC/ExPEC strains. The potential for cross-host transmission is real and can be traced back to contact with poultry meat and eggs contaminated with APEC [61]. Humans, unknowingly harbouring APEC/ExPEC strains symbiotically in their intestines, are at an increased risk of ExPEC infections, with BSIs being a prime concern [61, 62]. Given this scenario, gaining insights into APEC/ExPEC infection is paramount for the effective prevention and control of colibacillosis.

While the pivotal role of ProQ in stabilizing several sRNAs, including RyfA, has been documented in *E. coli* K-12 by Melamed et al. [64], the intricate interplay between ProQ and RyfA has yet to be thoroughly investigated. Our investigation thus offers comprehensive insight into the relationship between ProQ and the sRNA RyfA and contributes to a clearer understanding of the mechanisms governing APEC/ExPEC virulence and biofilm formation. Our findings illustrate the pronounced effect of ProQ deletion on APEC/ExPEC virulence, marked by a notable reduction in bacterial adhesion and survival. Similarly, the absence of the sRNA RyfA detrimentally impacted APEC/ExPEC virulence. The observed phenotypes of the mutant FY26Δ*ryfA* mirrored those of the mutant FY26Δ*proQ*, which were characterized by diminished colonization in vivo, reduced adhesiveness, and limited replication in macrophages. Aligning with our observations, a recent study highlighted the indispensability of RyfA for UPEC resistance to oxidative and osmotic stresses. This research also underscored the pivotal role of *ryfA* in promoting UPEC colonization in the urinary tract and ensuring its survival in macrophages [47]. This underscores the potential significance of the ProQ-RyfA interaction as a key modulator of APEC/ExPEC virulence. Our transcriptomic analysis further revealed that absence of the *proQ* gene led to the downregulated expression of APEC heat- and cold-shock proteins. This could compromise the bacterium’s adaptability to the multifaceted immune responses encountered during host tissue invasion. Moreover, the expression of several metabolic genes, such as the proline-associated *putA*, was also downregulated. Such alterations might hinder APEC’s growth and colonization in vivo, leading to diminished virulence.

Inactivation of the *ryfA* gene in the UPEC strain CFT073 led to diminished expression of type 1 and P fimbriae adhesins, subsequently causing decreased colonization within the mouse urinary tract [47]. In contrast, our transcriptomic investigations on the APEC *proQ* mutant revealed alterations in the transcription levels of various membrane proteins, such as *ompC*, *mltD*, *yagU*, and *ompW*. Notably, no transcription changes associated with fimbriae genes were identified. These observations prompt speculation that the role of ProQ in modulating APEC might be intrinsically linked with sRNA. Specifically, ProQ could be implicated in the translational regulation of proteins associated with adhesion via RyfA posttranscriptional regulation. This suggests that the observed changes in the APEC *proQ* mutant might be more pronounced at the protein translation level; we plan to investigate this hypothesis further in our subsequent studies. Further explorations are warranted to ascertain whether any protein alterations might be a consequence of downregulation of RyfA expression, which can be triggered by the deletion of *proQ* in APEC.

Bloodstream infections (BSIs) are severe systemic conditions that arise when pathogens penetrate the bloodstream. These infections can lead to bacteraemia or sepsis, and can potential inflicting severe harm to various organs. Given the rising prevalence and mortality associated with BSIs [65–67], the World Health Organization (WHO) designated sepsis as a Global Health Priority in 2017. Epidemiological findings indicate that the excessive use of antibiotics has paved the way for multidrug-resistant *E. coli* strains, which rank among the primary culprits of human BSIs. More specifically, aetiological research has identified the majority of *E. coli* strains causing BSI as ExPECs. The rates of rising drug resistance in these strains presents a significant challenge in clinical settings [60–62]. Concerningly, in recent years, we have seen an uptick in ExPEC BSI incidents, manifesting via a range of clinical symptoms. Without timely intervention, affected individuals may manifest conditions such as upper respiratory tract infections, pneumonia, and infections of the chest or abdominal cavity. Our current research underscores the pivotal role of ProQ in APEC/ExPEC-induced BSI and highlights the contribution of the sRNA RyfA to APEC/ExPEC BSI in a mouse sepsis model. Consequently, the interplay between ProQ and the sRNA RyfA might hold significant implications in the progression and management of APEC/ExPEC BSIs.

Biofilms are complex, protective microbial communities that play a pivotal role in bacterial life cycles [13, 68]. These communities form when bacteria adhere to various biological surfaces (e.g., meat products, oral cavities, skin, and agricultural goods) or abiotic surfaces (e.g., medical devices and drains) [15, 69]. Such formations can promote the survival of bacteria and augment their resistance against threats such as antibiotics, dehydration, ultraviolet radiation, and cleaning measures. The persistence of bacterial biofilms exacerbates the vulnerability of humans to infections. Overreliance on antibiotics and routine disinfectants in food processing can further amplify resistance among these pathogens, making their elimination especially challenging when they bind to food surfaces [70]. Alarmingly, the consumption of contaminated food leads to 2 billion infections and 1 million deaths globally each year [71, 72]. Our findings revealed that the biofilm produced by mutant FY26Δ*proQ* was considerably less robust than its WT FY26 counterpart, underscoring ProQ’s significance in biofilm genesis. This aligns with previous findings [38]. Moreover, since its discovery in 2001, the sRNA RyfA has been associated with *E. coli* biofilm development [45, 73]. We identified a direct interaction between the sRNA RyfA and ProQ, further illuminating the nexus between ProQ and APEC/ExPEC biofilm creation.

APEC strains derived from chicken meat have zoonotic potential, and they pose a risk in terms of human urinary tract infections [74]. The formation of biofilms by APEC/ExPEC on food product surfaces notably augments their resistance to environmental stresses. This escalation in resistance consequently amplifies the threat of ExPEC BSIs in humans due to foodborne transmission. To develop effective strategies that mitigate the threats of foodborne APEC/ExPEC, particularly in poultry products and their processing environments, a deep understanding of the molecular mechanisms governing ExPEC biofilm formation is essential. Our transcriptomic analysis revealed a marked downregulation in the transcription of RyfA in the *proQ* mutant strain. Intriguingly, the anticipated secondary structure of the sRNA RyfA aligns with the structural prerequisites essential for the binding of ProQ [33]. The binding of ProQ is posited to both stabilize the RyfA transcripts and increase its transcription levels. It is well established that sRNA-binding proteins modulate global bacterial gene expression via multiple routes. First, they facilitate translation by revealing the ribosome-binding site on the sRNA’s corresponding target mRNA. Concurrently, they shield the sRNAs target mRNA from exonucleolytic degradation, ensuring its stability [28]. However, the intricate posttranscriptional regulatory dynamics underlying the ProQ-RyfA interaction, especially in the context of global bacterial gene expression, merit deeper examination.

This study is an inaugural exploration that details the interaction between the RBP ProQ and the sRNA RyfA. We observed a pronounced downregulation of RyfA transcription following deletion of the *proQ* gene in APEC/ExPEC strains. EMSAs showed that ProQ directly associates with the sRNA RyfA, which features a predicted binding site at its 3′-end that is characterized by a stem‒loop tethered to a single-stranded poly(U) tail consisting of four paired units. Consequently, the ProQ-RyfA interaction is pivotal in determining the pathogenicity and biofilm-forming capability of APEC/ExPEC.

### Supplementary Information

**Additional file 1. List of bacterial strains, plasmids, and sequences of PCR primers employed in this research. **

**Additional file 2. Examination of lesions in chicks post-infection with the APEC strains WT FY26, FY26Δ*proQ*, and FY26C*proQ*** **at 24 hpi.** Chickens inoculated with PBS served as the negative control. (**A**) Presentation of hallmark symptoms of avian colibacillosis; affected chickens displayed neck retraction and reduced mobility, with several fatalities noted. (**B**) No evidence of colibacillosis-associated lesions such as air sacculitis, pericarditis, or perihepatitis was observed in chickens at 24 hpi with any of the APEC strains. (**C**) Histopathological assessment of organ-specific alterations in APEC-infected chickens revealed pulmonary congestion and haemorrhage coupled with hepatosplenomegaly in the groups infected with WT FY26, the mutant FY26Δ*proQ*, and the complemented strain FY26C*proQ*.

**Additional file 3. Pathological assessment of lesions in chicks post-infection with the APEC strains WT FY26, FY26Δ*proQ*, and FY26C*proQ*** **at 48 hpi.** Chickens inoculated with PBS served as the negative control. At 48 hpi, there was no evidence of colibacillosis-associated lesions such as air sacculitis, pericarditis, or perihepatitis in any of the infected groups.

**Additional file 4.** **Transcriptomic assessment of ProQ’s regulation of the expression of numerous genes within the APEC strain FY26.**

**Additional file 5. Critical role of RyfA in APEC/ExPEC virulence**. (**A**) The mortality of chickens administered WT FY26, the mutant FY26Δ*ryfA*,and the complemented strain FY26C*ryfA* were assessed. Survival rates were monitored up to 7 dpi. Chickens that received PBS injection served as a negative control. (**B**) In vivo colonization assessment highlighted the impact of RyfA deletion on the APEC strain FY26. (**C**) Examination of pathological alterations in the lung tissues of infected chickens at 24 hpi. Depicted are lung lesions from chickens infected with (a) WT FY26, (b) FY26Δ*ryfA*, and (c) FY26C*ryfA*. (d) A lung from a PBS-inoculated chicken. (**D**) Determination of the crucial role of RyfA in APEC/ExPEC BSI using a murine sepsis model. Survival rates were measured for mice infected with WT FY26, FY26Δ*ryfA*, andFY26C*ryfA*. (**E**) Investigation of the colonization efficacy of WT FY26, FY26Δ*ryfA*,and FY26C*ryfA* in mouse lungs and liver at 24 hpi. (**F**) Evaluation of pathological changes in the lungs and liver of mice exposed to WT FY26, FY26Δ*ryfA*,and FY26C*ryfA*. Illustrated are lung lesions from mice infected with (a) WT FY26, (b) FY26Δ*ryfA*,and (c) FY26C*ryfA*. (d) A lung from a PBS-administered mouse; liver lesions from mice exposed to (e) WT FY26, (f) FY26Δ*ryfA*, and (g) FY26C*ryfA*, with (h) a liver from a PBS-administered mouse.

**Additional file 6. Indispensable role of RyfA in the adhesion and intracellular survival of APEC/ExPEC.** (**A**) Assessment of adhesion to HEp2 cells by WT FY26, the mutant FY26Δ*ryfA*, and the complemented strain FY26C*ryfA*. (**B**) Evaluation of adhesion to DF-1 cells for the same strains. (**C**) Investigation into the role of the sRNA RyfA in promoting APEC intracellular persistence within HD11 macrophages. (**D**) Immunofluorescence analysis highlighting the impact of RyfA deletion on APECintracellular proliferation in HD11 cells. (**E**) The intracellular growth rates of WT FY26, FY26Δ*ryfA*, and FY26C*ryfA* in HD11 macrophages were monitored at 8 hpi. Note: Scale bar = 10 μm.

## Abbreviations

APEC: avian pathogenic *Escherichia coli*
ExPEC: extraintestinal pathogenic *Escherichia coli*
sRNA: small RNA
InPEC: intestinal pathogenic *Escherichia coli*
UPEC: uropathogenic *Escherichia coli*
NMEC: neonatal meningitis *Escherichia coli*
BSI: bloodstream infection
