# Supplementary figures and images for: ProQ binding to small RNA RyfA promotes virulence and biofilm formation in avian pathogenic Escherichia coli
Source: Vet Res. 2023 Nov 22;54:109. doi: 10.1186/s13567-023-01241-2 (PMC10664665; doi:10.1186/s13567-023-01241-2)

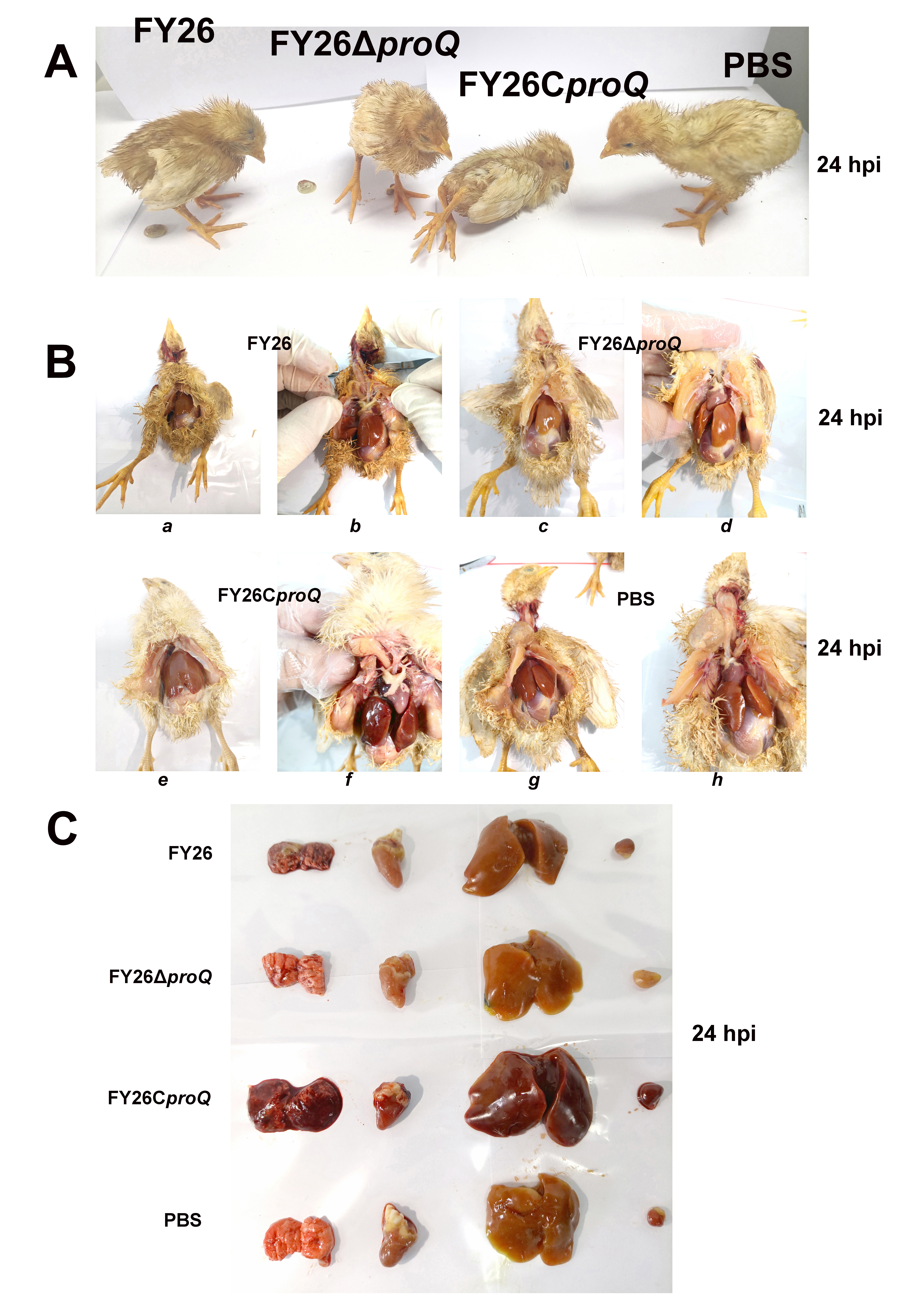

Supplement: Supplementary file 2 — Additional file 2. Examination of lesions in chicks post-infection with the APEC strains WT FY26, FY26ΔproQ, and FY26CproQ at 24 hpi. Chickens inoculated with PBS served as the negative control. (A) Presentation of hallmark symptoms of avian colibacillosis; affected chickens displayed neck retraction and reduced mobility, with several fatalities noted. (B) No evidence of colibacillosis-associated lesions such as air sacculitis, pericarditis, or perihepatitis was observed in chickens at 24 hpi with any of the APEC strains. (C) Histopathological assessment of organ-specific alterations in APEC-infected chickens revealed pulmonary congestion and haemorrhage coupled with hepatosplenomegaly in the groups infected with WT FY26, the mutant FY26ΔproQ, and the complemented strain FY26CproQ. [file 13567_2023_1241_MOESM2_ESM.jpg]

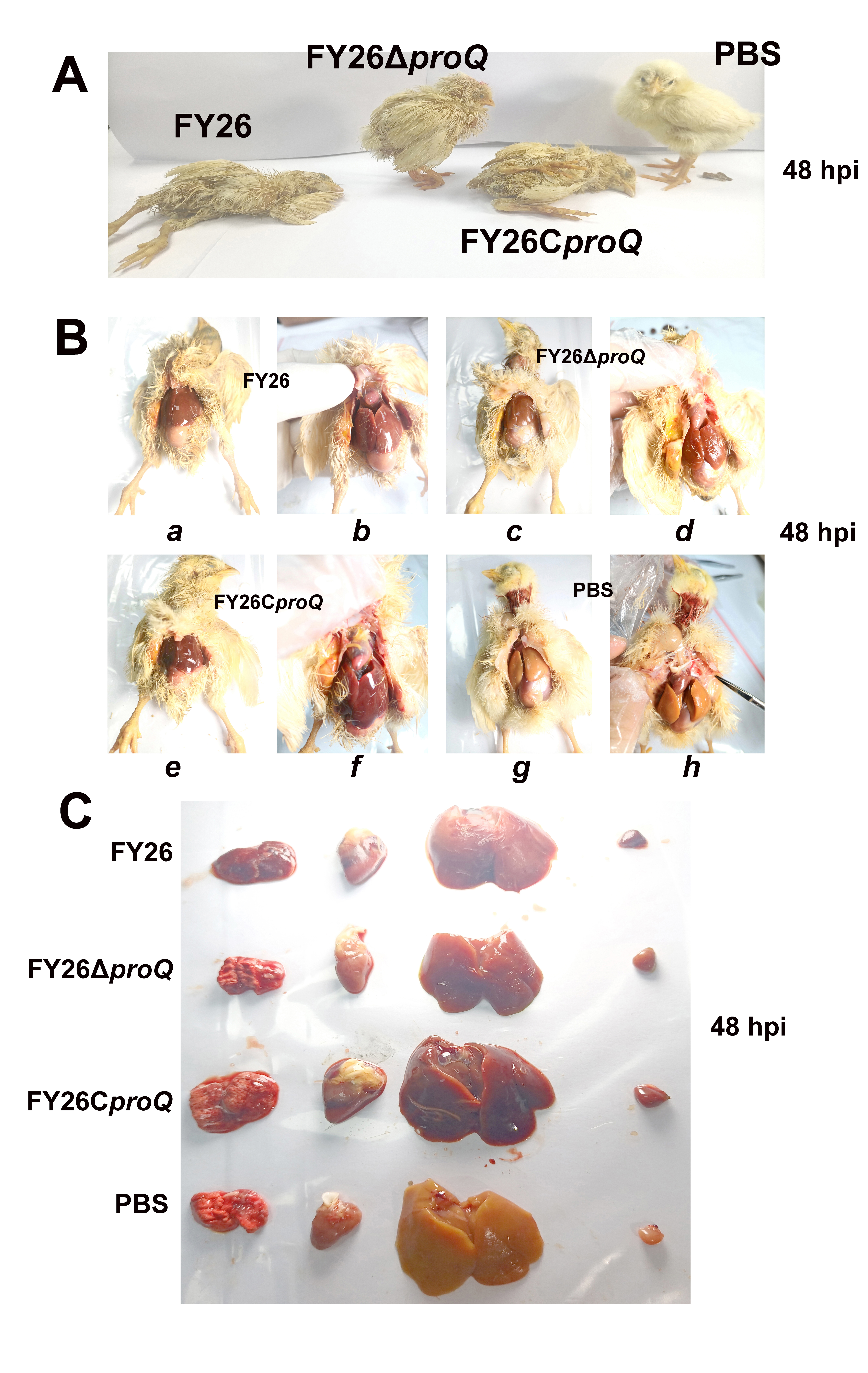

Supplement: Supplementary file 3 — Additional file 3. Pathological assessment of lesions in chicks post-infection with the APEC strains WT FY26, FY26ΔproQ, and FY26CproQ at 48 hpi. Chickens inoculated with PBS served as the negative control. At 48 hpi, there was no evidence of colibacillosis-associated lesions such as air sacculitis, pericarditis, or perihepatitis in any of the infected groups. [file 13567_2023_1241_MOESM3_ESM.jpg]

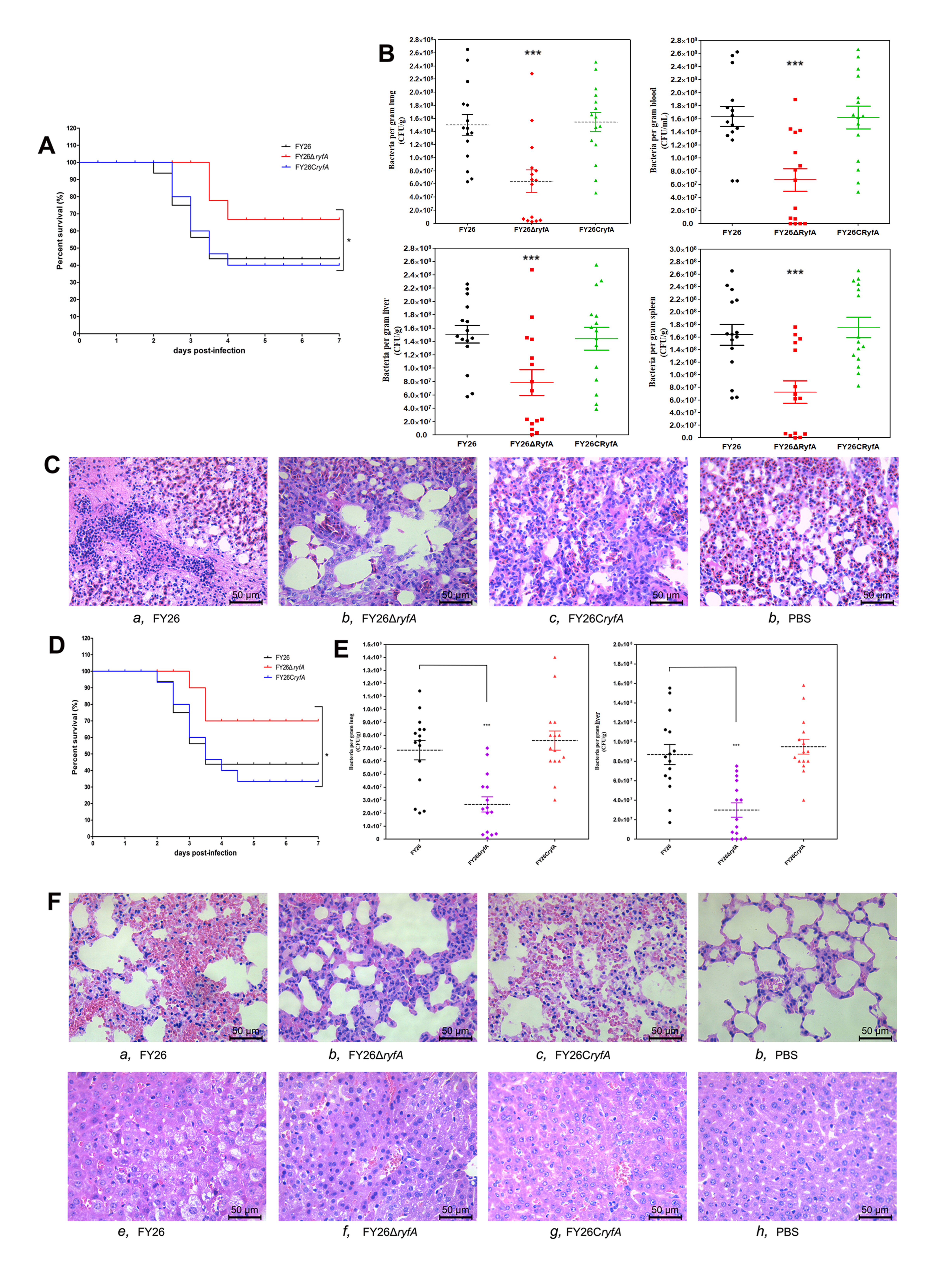

Supplement: Supplementary file 5 — Additional file 5. Critical role of RyfA in APEC/ExPEC virulence. (A) The mortality of chickens administered WT FY26, the mutant FY26ΔryfA,and the complemented strain FY26CryfA were assessed. Survival rates were monitored up to 7 dpi. Chickens that received PBS injection served as a negative control. (B) In vivo colonization assessment highlighted the impact of RyfA deletion on the APEC strain FY26. (C) Examination of pathological alterations in the lung tissues of infected chickens at 24 hpi. Depicted are lung lesions from chickens infected with (a) WT FY26, (b) FY26ΔryfA, and (c) FY26CryfA. (d) A lung from a PBS-inoculated chicken. (D) Determination of the crucial role of RyfA in APEC/ExPEC BSI using a murine sepsis model. Survival rates were measured for mice infected with WT FY26, FY26ΔryfA, andFY26CryfA. (E) Investigation of the colonization efficacy of WT FY26, FY26ΔryfA,and FY26CryfA in mouse lungs and liver at 24 hpi. (F) Evaluation of pathological changes in the lungs and liver of mice exposed to WT FY26, FY26ΔryfA,and FY26CryfA. Illustrated are lung lesions from mice infected with (a) WT FY26, (b) FY26ΔryfA,and (c) FY26CryfA. (d) A lung from a PBS-administered mouse; liver lesions from mice exposed to (e) WT FY26, (f) FY26ΔryfA, and (g) FY26CryfA, with (h) a liver from a PBS-administered mouse. [file 13567_2023_1241_MOESM5_ESM.jpg]
