# Supplementary material for: ProQ binding to small RNA RyfA promotes virulence and biofilm formation in avian pathogenic Escherichia coli
Source: Vet Res. 2023 Nov 22;54:109. doi: 10.1186/s13567-023-01241-2 (PMC10664665; doi:10.1186/s13567-023-01241-2)
Supplement: Supplementary file 4 — Additional file 4. Transcriptomic assessment of ProQ’s regulation of the expression of numerous genes within the APEC strain FY26. [file 13567_2023_1241_MOESM4_ESM.docx]

**1. Bacterial strains and plasmids used in this study.**

| **Bacterial strains and plasmids** | **Genotype or relevant characteristics** |
| --- | --- |
| **Bacterial strains** |  |
| *E. coli* DH5α | Plasmid propagation strain |
| *E. coli* BL21 | Protein expression strain |
| FY26 | O2:K1; ST 95; phylogroup B2 |
| FY26Δ*proQ* | *proQ* operon deletion in FY26 |
| FY26Δ*ryfA* | *ryfA* deletion in FY26 |
| FY26C*proQ* | FY26Δ*proQ* with plasmid pSTV28-*Ptac-proQ* |
| FY26C*ryfA* | FY26Δ*ryfA* with plasmid pSTV28-*ryfA* |
| FY26Δ*proQ-*Cp28 | FY26Δ*proQ* with the original plasmid pSTV28 |
| FY26Δ*ryfA-*Cp28 | FY26Δ*ryfA* with the original plasmid pSTV28 |
| FY26-*gfp* | FY26 with plasmid pUC19-*Ptac*-GFP |
| FY26Δ*proQ-gfp* | FY26Δ*proQ* with two compatible plasmids pSTV28-*Ptac-proQ* and pUC19-*Ptac*-GFP |
| FY26Δ*ryfA*-*gfp* | FY26Δ*ryfA* with plasmid pUC19-*Ptac*-GFP |
| FY26C*proQ*-*gfp* | FY26C*proQ* with two compatible plasmids pSTV28-*Ptac-proQ* and pUC19-*Ptac*-GFP |
| FY26C*ryfA*-*gfp* | FY26C*ryfA* with two compatible plasmids pSTV28-*ryfA* and pUC19-*Ptac*-GFP |
| FY26Δ*proQ-*Cp28-*gfp* | FY26Δ*proQ* with two compatible plasmids for the original pSTV28 and pUC19-Ptac-GFP |
| FY26Δ*ryfA-*Cp28-*gfp* | FY26Δ*ryfA* with two compatible plasmids for the original pSTV28 and pUC19-Ptac-GFP |
|  |  |
| ***Plasmids*** |  |
| pSTV28 | A medium-copy plasmid |
| pSTV28*-Ptac-proQ* | pSTV28 carrying the complete *proQ* gene and upstream/downstream DNA (artificially added Ptac promoter and rrnB terminator sequence) |
| pSTV28*-ryfA* | pSTV28 carrying *ryfA* coding region and its putative promoter |
| pCP20 | encodes FLP recombinase for removal of resistance cassette |
| pKD46 | λ-Red recombinase expression |
| pUC19-*Ptac*-GFP | pUC19 plasmid carrying the *Ptac*-GFP gene |
| pET28a | Kan, F1 origin, His tag |
| pET28a-*proQ* | pET28a carrying proQ gene |

**2. Oligonucleotide sequences used as PCR primers.**

| **Primers** | **Sequence (5'-3')** |
| --- | --- |
| **General PCR** |  |
| pSTV28-*proQ* |  |
| pSTV28-*ryfA*-F | CGAATTC CAGGCAATAAAAAACCGCCGAATCT |
| pSTV28-*ryfA*-R | CGTGGATCCCAGGTGAAAAAAGGGGAAAAAGGTA |
| **For Deletion^a^** |  |
| Del-*proQ*-F | CTGTTCATGCCTGCGCTTGTTGGCTACGTCCGTTGTAATCAGGAAATTTC GTGTAGGCTGGAGCTGCTTC |
| Del-*proQ*-R | CTAACGCGGTAAGCCTAAAAAACATGTTCATGCCTGGCCCGGCCTCCGTTCATATGAATATCCTCCTTAG |
| Del-*ryfA*-F | TTGTCAAGTAAAAGAGAGTTATTATTGTTCTGTTAGTGTATTATCCACTGTGTAGGCTGGAGCTGCTTC |
| Del-*ryfA*-R | CGTCGCTGGGGTGGTTTTTTGCGTGACTTTAAACAGAACCGGATAATTTCATATGAATATCCTCCTTAG |
| **For RT-PCR** |  |
| *ryfA*-qPCR-F | ATTGTTTCTGGTGCGCTGTT |
| *ryfA*-qPCR-R | CGGGCTTACTTGCTGTTCTG |
| *yeeE*-qPCR-F | ATCACCACCCAGAGCGTTAT |
| *yeeE* -qPCR-R | TGAGGGATTGATCGGCAGTT |
| *proQ*-qPCR-F | GAAGGTGAAAGCGGGTCAAA |
| *proQ*-qPCR-R | CGCCGTCTTTGGTGATTTCT |
| *ppnP -qPCR-F* | ACCGACTGGCAAGTGTATGA |
| *ppnP -qPCR-R* | ATAAGAGGTGGGTTCGGCAA |
| *cysA*-qPCR-F | GATAACGATCCGCCAGATGC |
| *cysA*-qPCR-R | GGACCGTAAAGTCGGTTTCG |
| *cysW* -qPCR-F | GACACGTCACGAAGATGGTG |
| *cysW* -qPCR-R | TGGTTGCTGGTCTGGTGTAT |
| *cysP*-qPCR-F | CAGTTTGTCGCCACCGTTAT |
| *cysP*-qPCR-R | GCTGGTTGCTTCTCTGCTAC |
| *cysN*-qPCR-F | TATGCAGCGATGAGAGCTGA |
| *cysN*-qPCR-R | TTTCTGACCTGTGGTAGCGT |
| *cysD*-qPCR-F | AAGCGGTCACGGAAAGAGTA |
| *cysD*-qPCR-R | TGAAACAGGCGCTGAACAAA |
| *ompC*-qPCR-F | ATTTCAGACCTGCGAATGCC |
| *ompC*-qPCR-R | TCTGTAGATGGCGACCAGAC |
| *mltD*-qPCR-F | AGTCGCCGTCAAACATCTTG |
| *mltD*-qPCR-R | TTTGATCCTCACGCAACGTC |
| *yagU*-qPCR-F | GCGTTGTTTCCGCATTTGTT |
| *yagU*-qPCR-R | GGATCTGTCAGTCCCAACCA |
| *ompW*-qPCR-F | CGTTGCTGCCAGTAATTCCA |
| *ompW*-qPCR-R | TACGTCCAACAGAAGGTGCT |
| *ompX*-qPCR-F | CGACCTACAAACACGACACC |
| *ompX*-qPCR-R | AATCCAGGTGCCTACGTCAA |
| *cspE*-qPCR-F | TTACGTTTGCAGCAGAAGGG |
| *cspE*-qPCR-R | GCTGGCTCTCCCAAACAA |
| *cspD*-qPCR-F | ATAACACTGGCGTGATTGCC |
| *cspD*-qPCR-R | TGCCAAAGGGTTTGGTTTCA |
| *hspQ-qPCR-F* | TGGATGGCTGTTCAGGATGT |
| *hspQ*-qPCR-R | AGGATGATAACGGCCTACCG |
| **For Northern blot** |  |
| *RyfA-Bio* | TGCGAAGACCGCATCACGAC |
| *5s-Bio* | ATGGGGTCAGGTGGGACCACCGCGCTACTGC |
|  |  |

**3. Complementary *proQ* sequence for DNA synthesis**

GTATAATGTGTGGAATTGTGAGCGGATAACAATTTCACACAGAAACAGCCAGTCCGTTTAGGTGTTTTCACGAGCACTTCACCAACAAGGACCATAGCATATGGAAAATCAACCTAAGTTGAATAGCAGTAAAGAAGTAATCGCGTTTCTGGCCGAACGTTTTCCCCACTGTTTCAGTGCGGAAGGTGAAGCGCGTCCGCTGAAAATCGGTATTTTTCAGGATTTGGTCGATCGTGTTGCTGGGGAAATGAACCTGAGCAAAACGCAATTGCGATCCGCTTTACGTCTCTACACTTCGAGCTGGCGTTATCTTTACGGTGTTAAACCCGGCGCAACGCGTGTCGATCTTGACGGCAACCCATGCGGTGAGCTGGACGAGCAACATGTAGAGCATGCTCGCAAGCAGCTTGAAGAAGCGAAAGCGCGTGTTCAGGCACAGCGTGCTGAACAGCAAGCGAAAAAACGCGAAGCTGCCGCAACTGCTGGTGAGAAAGAAGACGCACCGCGCCGCGAACGCAAGCCACGTCCGACTACGCCACGCCGCAAAGAAGGCGCTGAACGTAAACCTCGTGCGCAAAAGCCGGTAGAGAAAGCGCCAAAAACAGTAAAAGCACCTCGCGAAGAACAGCACACCCCGGTTTCTGACATTTCAGCTCTGACTGTCGGACAAGCCCTGAAGGTGAAAGCGGGTCAAAACGCGATGGATGCCACCGTATTAGAAATCACCAAAGACGGCGTCCGCGTCCAGCTGAATTCGGGTATGTCTTTGATTGTGCGCGCAGAACACCTGGTGTTCTGACGGCAGTAGCGCGGTGGTCCCACCTGACCCCATGCCGAACTCAGAAGTGA

**4. P_tac_-GFP-terninator sequence for DNA synthesis**

CCGGAATTCTCGGCTCGTATAATGTGTGGAATTGTGAGCGGATAACAATTTCACACAGGAAACAGCCAGTCCGTTTAGGTGTTTTCACGAGCACTTCACCAACAAGGACCATAGCATATGGTGAGCAAGGGCGAGGAGCTGTTCACCGGGGTGGTGCCCATCCTGGTCGAGCTGGACGGCGACGTAAACGGCCACAAGTTCAGCGTGTCCGGCGAGGGCGAGGGCGATGCCACCTACGGCAAGCTGACCCTGAAGTTCATCTGCACCACCGGCAAGCTGCCCGTGCCCTGGCCCACCCTCGTGACCACCCTGACCTACGGCGTGCAGTGCTTCAGCCGCTACCCCGACCACATGAAGCAGCACGACTTCTTCAAGTCCGCCATGCCCGAAGGCTACGTCCAGGAGCGCACCATCTTCTTCAAGGACGACGGCAACTACAAGACCCGCGCCGAGGTGAAGTTCGAGGGCGACACCCTGGTGAACCGCATCGAGCTGAAGGGCATCGACTTCAAGGAGGACGGCAACATCCTGGGGCACAAGCTGGAGTACAACTACAACAGCCACAACGTCTATATCATGGCCGACAAGCAGAAGAACGGCATCAAGGTGAACTTCAAGATCCGCCACAACATCGAGGACGGCAGCGTGCAGCTCGCCGACCACTACCAGCAGAACACCCCCATCGGCGACGGCCCCGTGCTGCTGCCCGACAACCACTACCTGAGCACCCAGTCCGCCCTGAGCAAAGACCCCAACGAGAAGCGCGATCACATGGTCCTGCTGGAGTTCGTGACCGCCGCCGGGATCACTCTCGGCATGGACGAGCTGTACAAGTAACGGCAGTAGCGCGGTGGTCCCACCTGACCCCATGCCGAACTCAGAAGCTTGGG
